# Microbial Inactivation and Quality Preservation of Chicken Breast Salad Using Atmospheric Dielectric Barrier Discharge Cold Plasma Treatment

**DOI:** 10.3390/foods10061214

**Published:** 2021-05-27

**Authors:** Eun Song Lee, Ye Jeong Jeon, Sea C. Min

**Affiliations:** Department of Food Science and Technology, Seoul Women’s University, Seoul 01797, Korea; pineles@naver.com (E.S.L.); dpwjd7510@naver.com (Y.J.J.)

**Keywords:** cold plasma, chicken salad, mixed vegetables, storage quality, sensory property

## Abstract

Microbiological safety of ready-to-eat foods is paramount for consumer acceptability. The effects of in-package atmospheric dielectric barrier discharge cold plasma (ADCP) treatment on the microbiological safety and quality of model chicken salad (CS) were investigated in this study. CS, packaged in a commercial polyethylene terephthalate container, was treated with ADCP at 24 kV for 2 min. The inactivation of indigenous mesophilic bacteria, *Salmonella*, and Tulane virus in CS; growth of indigenous mesophilic bacteria and *Salmonella* in CS; and quality of CS during storage at 4 °C were then investigated. ADCP inactivated indigenous mesophilic bacteria, *Salmonella,* and Tulane virus by 1.2 ± 0.3 log CFU/g, 1.0–1.5 ± 0.2 log CFU/g, and 1.0 ± 0.1 log PFU/g, respectively. Furthermore, it effectively retarded the growth of the microorganisms, while not significantly affecting the color of chicken, romaine lettuce, and carrot, and the antioxidant capacity of all vegetables throughout storage at the tested temperatures (*p* > 0.05). The color, smell, and appearance of all vegetables evaluated on day 0 were not significantly different in the sensory test, regardless of the treatment (*p* > 0.05). Collectively, ADCP treatment effectively decontaminates packaged CS without altering its quality-related properties.

## 1. Introduction

Ready-to-eat (RTE) foods, consumed without any further processing or preparations, are popular due to their availability, affordability, and palatability [1]. Packaged fresh-cut salad is among the most frequently consumed food products because of its freshness and convenience [2]. Many RTE salad products are a mixture of fresh vegetables and processed foods, such as chicken breast or salmon. However, there is a possibility of cross-contamination during mixing and packaging, which is a cause of RTE-associated food poisoning [3]. Most mixed salad products are consumed without further cooking, and thus the ability of conventional microbial decontamination method, including washing with chlorine solution, to prevent food poisoning is limited. Therefore, it is necessary to develop novel microbial inactivation approaches to enhance microbial safety of RTE salad products, while preserving the product quality attributes.

Cold plasma (CP) treatment inactivates microorganisms by generating various reactive species, free radicals, electrons, and ultraviolet photons [4,5]. Reactive oxygen and nitrogen species in atmospheric CP can break the chemical bonds of microbial cell membranes by colliding with the cell membrane [6]. In particular, reactive oxygen species (ROS) disrupts C–O, C–N, and C–C bonds that play an important role in the structure of peptidoglycans, thereby destroying the microbial cell wall and causing oxidative damage to the cell membrane and intracellular components. Additionally, ROS accumulates inside the cell to induce apoptosis [7,8]. Lipid peroxides produced by reactive species bind to DNA to form covalent adducts, which induce mutations and base-pair substitutions to cause microbial inactivation [9]. CP treatment using high voltage also causes accumulation of electrical charges on the bacterial cell membrane, which can cause irreversible breakdown of the transmembrane potential and subsequent bacterial inactivation [10]. Furthermore, UV photons in CP can also inactivate microorganisms by reacting with bases in the DNA strand to disrupt their ability to replicate [8].

Among the different CP treatments, atmospheric dielectric barrier discharge cold plasma (ADCP) treatment has recently garnered attention, as it can be applied to food products after packaging to prevent cross-contamination and has the potential to scale up for industrial application [6]. Among different plasma-forming gases, air, the least expensive gas, results in relatively high efficiency in microbial inactivation in foods, which is critical for the application of atmospheric CP treatment on an industrial scale [6]. However, the challenges for adoption of atmospheric CP treatment by the industry include the development of the treatment system compatible to current production lines, process stability, and regulatory approval [11]. Min et al. [12] reported that ADCP treatment (47.6 kV for 5 min) reduced indigenous microorganisms in romaine lettuce by 1.1 log CFU/g. Ziuzina et al. [13] reported that atmospheric CP treatment (80 kV, 5 min) inactivated *Salmonella* on iceberg lettuce by 2.4 log CFU/sample. Similarly, Roh et al. [14] reported that ADCP treatment (38.7 kV for 3.5 min) decreased the number of *Salmonella* on cooked chicken breast cubes (CBCs) by approximately 1.6 log CFU/cm^2^. However, no previous studies have reported the effects of in-package CP treatment on the storage quality of mixed chicken salad products. Thus, the objectives of this study were to determine the effects of ADCP treatment on the inactivation of *Salmonella* and Tulane virus (TV) in model chicken salad (CS), sensory properties of CS, growth of indigenous bacteria and *Salmonella*, and color and antioxidant capacity of CS during storage at 4 °C. The ADCP treatment conditions were also determined while investigating the effects of treatment time and shaking the CS containers during ADCP treatment on the efficacy of *Salmonella* inactivation by ADCP treatment.

## 2. Materials and Methods

### 2.1. Materials

Romaine lettuce, red cabbage, and carrot were purchased from a local grocery store. Raw chicken breast was purchased from Nobrand (Gunsan, Korea) and stored at –20 °C until further use. Whey protein isolate (WPI), used as a coating base material, was obtained from Davisco Foods International (Le Sueur, MN, USA).

### 2.2. Chicken Salad Preparation

The CS used in the current study consisted of romaine lettuce, red cabbage, carrot, and CBCs. Using sterile knives and a pair of scissors, the romaine lettuce, red cabbage, and carrot were cut into pieces that were approximately 40 × 50 mm, 10 × 45 mm, and 45 × 2 × 2 mm each, respectively. In the experiments determining the effects of ADCP treatment on the inactivation of *Salmonella* and TV, the vegetable pieces were immersed in 300 mL of sodium hypochlorite solution (300 ppm) and rubbed for 1 min. Thereafter, the vegetable pieces were washed 5 times with distilled water and air-dried in a biohazard hood at 24 ± 3 °C and 30 ± 2% relative humidity (RH) for 1 h. In the experiments determining the effects of ADCP treatment on the inactivation of indigenous bacteria, and CS quality and sensory properties, vegetable pieces were rubbed gently in sterile distilled water for approximately 10 s, followed by rinsing with sterile distilled water. The vegetable pieces were then dried in a biohazard hood for 1 h.

CBCs were prepared as described by Roh et al. [14]. Briefly, frozen raw chicken breast (1 kg) was placed in a household pot and boiled for 15 min at 99 to 100 °C. The boiled CBCs were obtained by cutting the meat into 1.5 cm × 1.5 cm × 1.5 cm pieces (3.5 g each) using a razor sterilized with 70% (*v/v*) ethanol in a biohazard hood. Subsequently, the CBCs were coated on all sides with a WPI coating solution, which was prepared as previously described [15]. Briefly, the coating solution was prepared by heating 10% (*w*/*w*) WPI solution in a 90 °C water bath for 30 min, and then mixed with glycerol (5%, *w*/*w*), cooled at 23 ± 2 °C, and degassed using a vacuum pump. The CBCs were immersed in the WPI coating solution (20 mL) for 1 min to coat all sides of CBCs and then dried in a biohazard hood for 2 h. The coating process was repeated 3 times.

The final CS (18 g) consisted of 3 pieces of romaine lettuce, 6 pieces of red cabbage, 8 pieces of carrot, and 3 CBCs. It was packaged in a commercial polyethylene terephthalate (PET) container with a separate lid (140 mm × 100 mm × 30 mm; thickness: 0.29 ± 0.01 mm; volume: 235 mL; DL-601; Dongyang D & P, Korea) for subsequent ADCP treatment. The headspace volume in the container was ~165 mL. Before use, the PET containers were sterilized with 70% ethanol, washed with distilled water, and dried in a biohazard hood. The packaged CS is referred to as the CS sample.

### 2.3. Microbial Inoculum Preparation

The *Salmonella* strains used in the current study were *S*. Enteritidis (CCARM 8040), *S*. Montevideo (CCARM 8052), and *S*. Typhimurium DT 104. *S*. Enteritidis and *S*. Montevideo were obtained from the Culture Collection of Antimicrobial Resistant Microbes (Seoul Women’s University, Seoul, Korea), and *S*. Typhimurium DT 104 was obtained from the Agricultural Biotechnology Culture Collection (Seoul National University, Seoul, Korea). Each *Salmonella* strain was pre-cultured twice in tryptic soy broth (BD, Franklin Lakes, NJ, USA) at 37 °C for 24 h. The cultured strains were centrifuged (3600× *g*, 5 min) and washed twice with 0.1% (*w*/*v*) peptone water. After washing, equal volumes of the strain suspensions were mixed to prepare the *Salmonella* cocktail (~9 log CFU/mL), and diluted with 0.1% peptone water to prepare an inoculum (~8 log CFU/mL). Bacterial concentration was then checked by culturing on xylose-lysine-deoxycholate agar (BD).

TV is used as a surrogate of human norovirus [16,17]. TV and monkey kidney cell line LLC-MK2 used in the current study were obtained from the Cincinnati Children’s Hospital Medical Center (Cincinnati, OH, USA) and Korea Cancer Center Hospital (Seoul, Korea), respectively. The growth medium was M199 medium (Gibco, Grand Island, NY, USA) supplemented with 10% (*v*/*v*) heat-inactivated fetal bovine serum (FBS; Gibco) and 1% (*v*/*v*) antibiotic–antimycotic solution (Cellgro Mediatech Inc., Herndon, VA, USA) [17]. The LLC-MK2 cells were cultured in a humidified incubator at 37 °C, with the atmosphere adjusted to 95% air and 5% CO_2_. Confluent LLC-MK2 cells were infected with TV, incubated at 37 °C for 1 h in the 5% CO_2_ atmosphere, and then 25 mL of M199 (10% FBS) was added. After 2 d, TV was harvested as the TV inoculum (~5 log plaque-forming unit (PFU)/mL) using three freeze–thaw cycles and centrifugation (3000× *g*, 10 min).

For microbial inoculation, each CS sample in an uncapped PET container was sprayed with 1 g of *Salmonella* or TV inoculum using a sterilized glass sprayer. The masses of the CS sample before and immediately after inoculation were used to verify the 1-g inoculation mass. After inoculation, the CS was dried in a biohazard hood for 2 h, and then used as a sample for analysis. The *Salmonella* and TV densities on dried CS were ~6 log CFU/g and ~3 log PFU/g, respectively.

### 2.4. In-Package Atmospheric Dielectric Barrier Discharge Cold Plasma Treatment

ADCP treatment was performed using a system using atmospheric air as a plasma-forming gas, which was described by Kim et al. [18]. In the system, plasma was formed between two aluminum electrodes (20 cm × 16 cm, AL6061; Kwang-lim Co. Ltd., Hwasung, Korea). A sheet of borosilicate (25 cm × 29 cm, 3.5-mm–thick), a dielectric barrier, was placed on the bottom electrode. The electrodes were connected to an alternating current power supply (max. 40 kV, 60 Hz). Voltage was measured using a high-voltage electrical probe (EP-50; Pulse Electronic Engineering Co., Ltd., Noda, Japan) and displayed using a digital oscilloscope (TDS-3012b Oscilloscope; Tektronix, Beaverton, OR, USA). The PET container with CS was positioned in the space between the upper electrode and the dielectric barrier. The distance between the PET container and the upper electrode was set at 0.5 cm. The ADCP treatment was conducted at 24 kV, at which no direct dielectric breakdown nor any color change occurred in CS according to our preliminary study [19]. Before treatment, the container was capped with its lid tightly and the capped area was taped (KS-S3166; KisanBio, Seoul, Korea) for hermetic sealing. To determine the effect of shaking of the container during treatment on the efficacy of microbial inactivation by the treatment, the container was shaken using insulated bars connected to a digital reciprocating shaker (Daihan Scientific Co., Ltd., Daejeon, Korea) and agitated at 300 rpm during treatment for 2 min. To evaluate the effect of treatment time on *Salmonella* inactivation by the ADCP treatment, the CS samples were treated for 1, 2, 3, 4, or 5 min without shaking the container. The CS samples prepared for the storage study were subjected to ADCP treatment for 2 min without shaking.

### 2.5. Microbial Analysis

For indigenous mesophilic aerobic bacteria and *Salmonella* analyses, the entire CS (18 g) in the PET container and 72 mL of 0.1% peptone water were placed in a stomacher bag (Whirl-Pak, 720 mL; Nasco, Fort Atkinson, WI, USA), which was then pummeled by a stomacher blender (Lab Blender Model 400; Seward Medical, London, UK) for 3 min at 230 rpm. The homogenized solution was diluted, spread on plate count agar and xylose-lysine-deoxycholate agar, and then incubated at 36 ± 1 °C for 48 and 24 h before colony counting. For TV analysis, the homogenate was passed through a 0.22-µm filter unit (Corning Inc., Corning, NY, USA) to obtain the viral solution. The viral titer was quantified using the LLC-MK2 plaque assay [17]. LLC-MK2 cells were seeded in a six-well plate (BD) at 2 × 10^5^ cells/well, and then 2.5 mL of M199 growth medium supplemented with penicillin G (100 U/mL), 0.5% agarose, 1% antibiotic–antimycotic solution, and 10% FBS was added. The cells in each well were infected with a diluted viral solution and incubated at a 5% CO_2_ atmosphere and 37 °C for 4 d. The cells and viruses were then fixed with 3% formaldehyde (Thermo Fisher Scientific, Waltham, MA, USA) and stained with 0.05% crystal violet (*w/v* in 10% ethanol) for plaque counting. The data are expressed as PFU/g.

### 2.6. Storage Study

The storage study was designed to determine the effect of ADCP treatment on the growth of indigenous mesophilic aerobic bacteria in CS; the color and antioxidant capacity of romaine lettuce, red cabbage, and carrot in CS; and the color of CBCs. CS samples in PET containers were prepared as described in Section 2.1—but without *Salmonella* inoculation. The samples were prepared with or without ADCP treatment, and stored at 4 °C for 0, 3, 5, 7, 10, or 14 d. *Salmonella*-inoculated CS samples, prepared as described in Section 2.1, were stored at 4 °C for 0, 3, 5, 7, 10, or 14 d, with or without ADCP treatment, to examine the effect of ADCP treatment on the growth of *Salmonella* during storage.

To measure the temperature and humidity during storage, individual samples were prepared by placing CS in PET containers with data loggers (8829S; AZ Instrument Corp., Taichung City, Taiwan). These CS samples were not subjected to the ADCP treatment. The temperature during storage was 4.4 ± 0.5 °C; the relative humidity in the packaging container was 99.8 ± 0.4%.

### 2.7. Color Measurement

The color was measured using a colorimeter (Minolta Chroma Meter CR-400; Minolta Camera Co., Osaka, Japan) with Illuminate D65, 2° standard observer, and a CIELAB scale. The colorimeter was calibrated using the white plate (Minolta calibration plate No. 14233126, Y = 87.4, x = 0.3174, and y = 0.3353). Two CS samples were analyzed on each day of sampling. Three CBCs were randomly chosen from each sample and one measurement was made per piece. Three spots on the romaine lettuce, red cabbage, and carrot were measured. The color difference (Δ*E*) was calculated using the following Equation.

(1) $$\Delta E=\sqrt{{\left(\Delta L^{*}\right)}^{2}+{\left(\Delta a^{*}\right)}^{2}+{\left(\Delta b^{*}\right)}^{2}}$$

### 2.8. Antioxidant Capacity Determination

The extraction procedure for determining the antioxidant capacity of romaine lettuce, red cabbage, and carrot in CS was performed as described by Liu et al. [20]. Briefly, each sample (5 g) was mixed with 10 mL of 80% ethanol, and homogenized using a homogenizer at 8000× *g* for 2 min. The homogenized solution was passed through a Whatman No. 2 filter paper (Whatman, Maidstone, Kent, UK). The filtrate was centrifuged at 10,000× *g* for 10 min, and the obtained supernatant was used as the samples to be analyzed. The antioxidant capacity was analyzed using assays to measure the 2,2-diphenyl-1-picrylhydrazyl (DPPH) scavenging activity and 2,2′-azino-bis(3-ethylbenzothiazoline-6-sulfonic acid) (ABTS) scavenging activity. These assays are frequently used for determining the antioxidant capacity of vegetables, such as salad products [20,21].

The DPPH scavenging activity was analyzed using the method of Blois [22]. For the analysis, DPPH (Sigma-Aldrich Co., St. Louis, MO, USA) was dissolved in 80% ethyl alcohol to a concentration of 0.32 mM. Thereafter, 100 µL of the DPPH solution was mixed with 10 µL of the sample to be analyzed. The mixture was left to react in the dark at 23 °C for 30 min. The absorbance of the sample, indicative of the radical scavenging activity, was measured at 517 nm (Spectra Max 250; Molecular Device, Sunnyvale, CA, USA).

The ABTS scavenging activity was analyzed using the method described by Re et al. [23]. Briefly, 10 mM potassium persulfate (Sigma-Aldrich Co.) and 10 mM ABTS (Sigma-Aldrich Co.) were mixed at a 2.6:7.4 ratio, and left to react in the dark for 24 h. The mixture was then diluted 10-fold using phosphate-buffered saline (pH 7.4; Sigma-Aldrich Co.) to prepare the working ABTS solution. The working ABTS solution (150 µL) was mixed with 50 µL of the sample to be analyzed and the mixture was left to react in the dark at 23 °C for 30 min. Finally, sample absorbance was measured at 734 nm (Spectra Max 250).

Two CS samples were analyzed on each day of sampling. All vegetables in each CS sample were used for determining the antioxidant capacity. The determination of antioxidant capacity involved three measurements per sample.

### 2.9. Sensory Test

The sensory test was performed after storing the untreated and ADCP-treated CS at 4 °C for 0 and 3 d. The storage time (3 d) was chosen because the shelf life of commercial CS at refrigerated temperature is commonly 3 d. The panelists for the sensory test were undergraduate and graduate female students (aged 20–29 years) of the Department of Food Science and Technology at Seoul Women’s University (Seoul, Korea). The panelists were initially screened for the consumption frequency of CS, with women consuming CS more than twice a month. The number of panelists was 40, which is larger than the number considered to be minimally required to make a preference sensory test valid [24,25]. The panelists participated in a 9-point intensity test (1 point: extreme dislike; 5 points: normal; 9 points: extreme like) to evaluate the appearance, color, and smell of CS. In addition, the panelists participated in a discrimination test, in which they were asked to choose one out of three samples, which displayed a difference [26]. Sensory tests were conducted without tasting the CS samples in the mouth. 

### 2.10. Statistical Analysis

The *Salmonella* inactivation experiment for determining the ADCP treatment conditions was repeated four times. For each replicate, two CS samples (two containers) were analyzed for each treatment, as described in Section 2.5. The storage study was repeated twice, and two CS samples were analyzed on each day of sampling to determine the number of microorganisms and the quality properties of CS for each replicate. One-way analysis of variance (ANOVA) was performed to analyze the differences between means using SPSS (ver. 23.0.0; IBM SPSS Inc., New York, NY, USA). When significant differences were observed, Tukey’s multiple range test was conducted, to analyze the means for significant difference determination (*α* = 0.05). A binomial test using XLSTAT (an add-in in Microsoft Excel, Paris, France) was performed to analyze significant differences in the sensory properties with and without the treatment (*α* = 0.05) [27].

## 3. Results and Discussion

### 3.1. Determination of ADCP Treatment Conditions

Table 1 presents the effect of treatment time on the inactivation of *Salmonella* in CS by ADCP at 24 kV. The efficacy of the 2-min treatment was significantly higher than that of the 1-min treatment (*p* < 0.05). However, the treatment time did not have a linear effect on *Salmonella* inactivation. ADCP treatments for 2, 3, 4, and 5 min resulted in approximately 1 log CFU/g reduction in *Salmonella* counts. Insignificant differences in the extent of *Salmonella* inactivation upon treatment time exceeding 2 min indicate that approximately 1 log CFU/g reduction is the highest reduction that can be achieved with the ADCP treatment, and the antimicrobial substances generated by ADCP, which were effective against *Salmonella* in CS, were maximally generated inside the packaged CS after a 2-min treatment. Lee et al. [19] reported that the optimum treatment time for in-package ADCP treatment at 24 kV was 3 min for inactivating *Salmonella* in ready-to-eat chicken breast cubes when 2.0, 2.5, and 3.0 min were tested. The difference in the best treatment times could be partially induced by the different sizes of empty space in the containers in the two studies. When both package volume and food volume were considered, the empty space in the container in the current study was likely smaller than that in the previous study. Reactive species in cold plasma effective against *Salmonella* could be formed at maximum concentrations faster in a smaller empty space, that is, in the container used in the current study.

The extent of *Salmonella* inactivation by the 2-min treatment, with and without shaking the CS containers during treatment, was 1.2 ± 0.1 and 1.0 ± 0.2 log CFU/g, respectively, suggesting that the shaking did not enhance the treatment inactivation efficacy. Nevertheless, an increase in the inactivation efficacy of ADCP treatment by shaking during CP treatment has been reported by previous studies [14,28]. In our preliminary study [14], shaking during ADCP treatment (38.7 kV for 3.5 min) increased the extent of *Salmonella* inactivation in a cooked chicken breast sample from 1.6 to 2.8 log CFU/cm^2^. The inactivation enhancement was thought to be associated with the rolling during shaking, which could facilitate the exposure of all contaminated areas to CP [14]. Nonetheless, in the current study, the shaking did not effectively roll or move the CS contents because of the geometry of the samples generated by stacked vegetable leaves. This would lead to the observed lack of improvement in *Salmonella* inactivation efficacy by shaking. The results suggest that, rather than promoting the motion of various reactive species in the headspace of the packaging container, the shaking promotes the movement of foods to allow a more effective exposure to reactive species, with a resultant potentiation of microbial inactivation. Based on this result, shaking was not applied to the treatments in the other experiments.

### 3.2. Tulane Virus Inactivation

**Figure A1:**
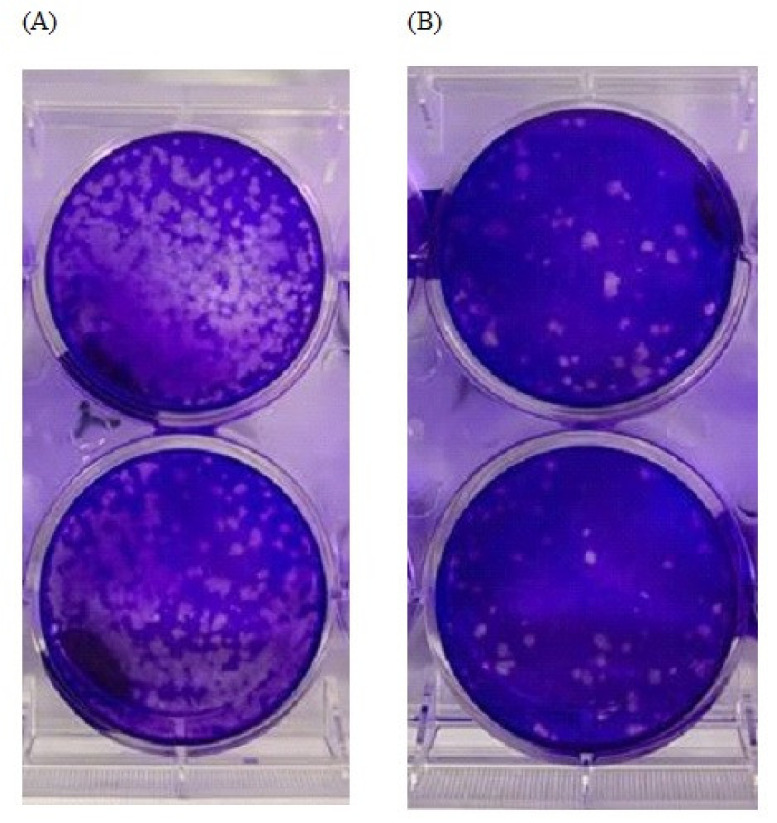
Plaque assay of Tulane virus on chicken salad without (**A**) and with (**B**) atmospheric dielectric barrier discharge cold plasma treatment.

The ADCP treatment at 24 kV for 2 min inactivated TV in CS by 1.0 ± 0.1 log PFU/g (Figure A1). A similar level of TV inactivation by CP treatment has been reported [16,17]. The ADCP treatment at 34.8 kV for 5 min inactivated TV in romaine lettuce by 1.3 ± 0.1 log PFU/g [17], and atmospheric plasma jet treatment at 549 W for 45 s with a pulse frequency of 47 kHz inactivated TV in blueberry by 1.5 log PFU/g [16]. CP treatment inactivates TV, as the reactive species and ultraviolet photons generated during CP treatment damage the viral capsid by breaking the polypeptide chains in the capsid structure [29,30], and react with the viral RNA encoding the surface protein, capsid protein, maturation protein, lysis protein, and replicase protein [16,31]. These observations imply that the ADCP treatment is effective in inactivating human norovirus contaminating salad products of mixed fresh vegetables and cooked meat.

### 3.3. Storage Study

#### 3.3.1. Microbial Growth during Storage

**Figure 1:**
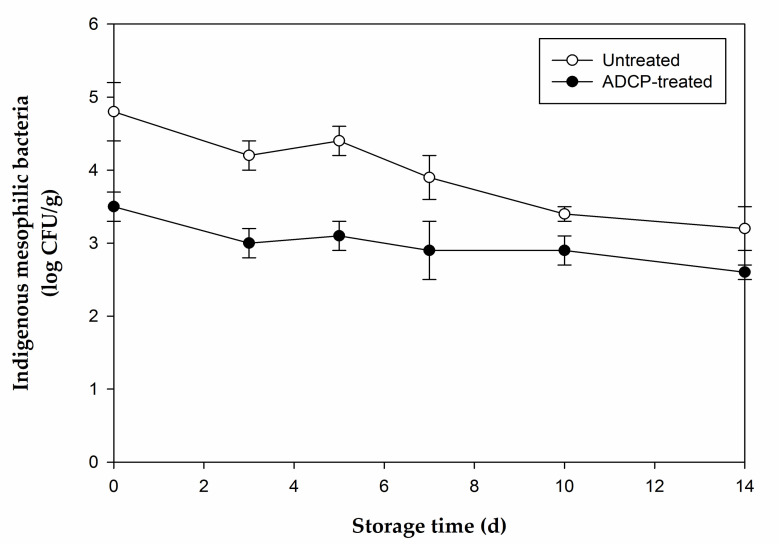
The effect of atmospheric dielectric barrier discharge cold plasma (ADCP) treatment on the growth of mesophilic aerobic microorganism in chicken breast salad during storage at 4 °C. Error bars represent standard deviation (*n* = 4).

The initial microbial count of mesophilic aerobic bacteria on CS was 4.8 ± 0.4 log CFU/g and was reduced to 3.5 ± 0.2 log CFU/g immediately after the ADCP treatment (Figure 1). The inactivation efficacy of ADCP treatment was similar to that of washing with sodium hypochlorite solution, which is the most common microbial decontamination method used for minimally processed foods, including fresh produce, resulting in a reduction of 1–2 log cycles [32]. Both untreated and treated samples exhibited a decreasing trend of microbial counts during storage at 4 °C for 14 d, whereas the levels of microbial count reduction (the difference between the counts of treated and untreated samples on each storage day) were not noticeably different (0.6–1.2 log CFU/g). If the ADCP treatment had a sublethal effect on the indigenous microorganisms on CS, cold storage would have further reduced the microbial cell population on ADCP-treated CS. As the levels of microbial count reduction were similar on each storage day, the ADCP treatment used in the current study most likely had a direct lethal effect rather than a sublethal effect on the microorganisms on CS. Post-treatment storage reportedly enhances the microbial inactivation efficacy of ADCP, as CP-generated reactive species diffuse inside the produce tissue during storage in a closed container [33].

**Figure 2:**
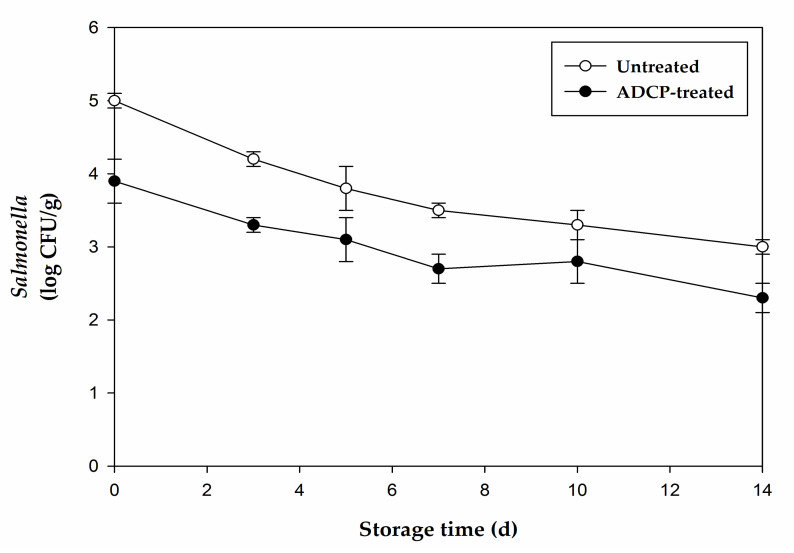
Effects of atmospheric dielectric barrier discharge cold plasma (ADCP) treatment on the growth of *Salmonella* in chicken breast salad during storage at 4 °C. Error bars represent standard deviation (*n* = 4).

The initial *Salmonella* inactivation on CS by the ADCP treatment was 1.0 ± 0.2 log CFU/g (Figure 2). The extent of *Salmonella* inactivation during storage was similar to that of indigenous bacterial inactivation. During storage at 4 °C for 14 d, the microbial counts of ADCP-treated CS were lower than those of untreated CS, by 0.7–1.5 log CFU/g.

#### 3.3.2. Color

The analysis results of the color of romaine lettuce, red cabbage, carrot, and CBCs in CS during storage following the ADCP treatment are presented in Table 2. During storage at 4 °C, none of the measured values (L *, *a* *, b *, and Δ*E*) of romaine lettuce, carrot, and CBCs were significantly affected by the ADCP treatment (*p >* 0.05).

These salad ingredients were thus suitable for treatment with ADCP. In contrast, the *L* *, *a* *, *b* *, and Δ*E* values for red cabbage during storage at 4 °C were significantly changed by the ADCP treatment from day 1. The color change of red cabbage is thought to be associated with a partial destruction of the pigment anthocyanin by CP [34], resulting in an increasing *L* * and *b* * value trend and a decreasing *a* * value trend during storage. As anthocyanin is present on food surface, it is prone to destruction by heat or oxygen radicals [35]. The ozone and radicals generated during ADCP treatment may directly react with anthocyanin, or secondary oxidizing agents, such as O2•− and •OH, and may induce the loss of anthocyanin [36]. Lacombe et al. [34] reported that an atmospheric CP jet treatment (549 W, 90 s) led to a significant reduction in the cyanidin 3-galactoside level in blueberry. The effects of ADCP on the color varied according to the product, indicating the need for treatment parameters tailored to each food product [37]. Furthermore, consequently, the production of RTE salad should be based on the selection of fruits and vegetables suitable for CP treatment.

#### 3.3.3. Antioxidant Capacity

The DPPH and ABTS radical scavenging activities of romaine lettuce, red cabbage, and carrot in CS are presented in Table 3. During storage at 4 °C, all samples exhibited a decreasing trend of antioxidant capacity with an increasing storage period, but no differences related to the ADCP treatment were observed. The results of the DPPH and ABTS assays for lettuce were different on day 0, irrespective of cold plasma treatment. This discrepancy might be because the DPPH assay, which detects the antioxidant activity of hydrophobic materials, more sensitively detected the decreased antioxidant activity of the antioxidant materials in lettuce than the ABTS assay, which measures the antioxidant activity of both hydrophilic and hydrophobic antioxidants [38]. Future research is needed to verify this by systematically conducting qualitative and quantitative analyses on antioxidants in lettuce.

The components of vegetables, such as vitamins and phenolics, are bioactive compounds that affect the antioxidant capacity, and they may react with the reactive species generated during CP treatment and become oxidized [4,39]. Nevertheless, studies have reported that ADCP treatment does not influence the antioxidant capacity of vegetables. Song et al. [39] stored lettuce after a microwave-powered CP treatment at 4 °C and 10 °C for 12 d, and observed no significant differences in the DPPH and ABTS levels between samples that were or were not CP treated (900 W, 10 min). Pasqual et al. [40] used ADCP (15 kV, 30 min) to treat radicchio and reported a lack of effect on the ABTS level. The observations of the current study confirm that the ADCP treatment disinfects vegetables without lowering their antioxidant activities.

#### 3.3.4. Sensory Evaluation

The sensory evaluation of untreated and ADCP-treated CS on days 0 and 3 during storage at 4 °C was performed using a 9-point scale (Table 4). The samples that were not stored showed no significant change in any sensory parameters. Similarly, in a discriminative test immediately after the ADCP treatment, no significant differences were observed. However, when the samples were stored at 4 °C for 3 d, a significant reduction in preference with respect to the color and appearance was noted (Table 4). The difference in the color of ADCP-treated and untreated CS stored for 3 d was attributed to the difference in color between the 3-d–stored red cabbage with and without ADCP, determined using a colorimeter (as presented in Table 2). The appearance of ADCP-treated CS had a lower score than that of untreated CS, and this could also be influenced by the color change in ADCP-treated red cabbage during storage.

For “smell”, no significant differences with storage were observed (*p >* 0.05). The reactive species formed after CP treatment may facilitate the oxidation and peroxidation of cell membranes in foods [41], which in turn might impart an off-taste. However, the amount of reactive species generated during the treatment in the current study was thought to not be substantial enough to affect the smell of CS.

These observations confirmed that the red cabbage is not a suitable CS vegetable to be subjected to ADCP. Hence, for the production of RTE salad with CP treatment, foodstuffs that are tolerant to the treatment should be selectively used.

## 4. Conclusions

The ADCP treatment conditions for CS packaged in a PET container were determined as 24 kV and 2 min; under these conditions, the ADCP treatment inactivated *Salmonella* on CS, reducing the counts by 1.0–1.5 ± 0.2 log CFU/g, and TV, reducing the counts by 1.0 ± 0.1 log PFU/g. During storage at 4 °C, the ADCP treatment led to the reduction in indigenous mesophilic bacteria and *Salmonella* counts on CBCs, suppressing their levels by 0.6–1.2 log CFU/g and 0.7–1.5 log CFU/g, respectively. At 4 °C, the color of romaine lettuce and carrot; the antioxidant capacity of romaine lettuce, red cabbage, and carrot; and the color of CBCs were not affected by the treatment. In the sensory evaluation, the ADCP treatment did not significantly affect the color, smell, and appearance of CS. However, after 3 d of storage at 4 °C, the color and appearance of ADCP-treated CS were less appealing to the panelists than untreated CS, and this was attributed to the color change of red cabbage, which was found susceptible to CP treatment. These observations indicate that ADCP treatment has the potential for decontaminating packaged CS, without altering its quality properties.

## Figures and Tables

**Table 1:** Effect of the treatment time of atmospheric dielectric barrier discharge cold plasma treatment on *Salmonella* inactivation.

Conditions		*Salmonella* (log CFU/g)	Microbial Reduction (log CFU/g)
Treatment Voltage	Treatment Time		
24 kV	1 min	5.5 ± 0.2	0.3 ± 0.1 b
	2 min	4.9 ± 0.2	1.0 ± 0.2 a
	3 min	5.0 ± 0.2	0.8 ± 0.2 a
	4 min	5.0 ± 0.2	0.9 ± 0.2 a
	5 min	4.9 ± 0.3	1.0 ± 0.2 a

The values are mean and standard deviation (*n* = 4). Different letters indicate significant difference between data in the same column (*p* < 0.05).

**Table 2:** Effects of atmospheric dielectric barrier discharge cold plasma (ADCP) treatment at 24 kV for 2 min on the color of salad during storage at 4 °C.

Content	Storage Time (d)	*L* *		*a* *		*b* *		Δ*E*	
		Untreated	ADCPT	Untreated	ADCPT	Untreated	ADCPT	Untreated	ADCPT
Romaine Lettuce	0	38.23 ± 2.05 ^b^	38.95 ± 3.79 ^b^	−16.10 ± 5.83 ^ab^	−16.82 ± 1.76 ^ab^	27.06 ± 4.80 ^a^	26.21 ± 3.96 ^a^	0	3.25 ± 1.29 ^a^
	3	39.60 ± 3.19 ^ab^	39.96 ± 2.91 ^ab^	−16.08 ± 1.33 ^a^	−16.25 ± 1.05 ^a^	26.02 ± 2.26 ^a^	26.88 ± 2.09 ^a^	2.65 ± 1.77 ^a^	3.17 ± 1.46 ^a^
	5	40.07 ± 2.82 ^ab^	41.35 ± 1.96 ^ab^	−16.46 ± 1.61 ^ab^	−16.88 ± 1.14 ^ab^	27.73 ± 1.24 ^a^	27.24 ± 1.81 ^a^	3.2 ± 2.19 ^a^	3.58 ± 1.5 ^a^
	7	41.66 ± 2.17 ^a^	41.12 ± 2.54 ^ab^	−16.68 ± 0.88 ^ab^	−16.36 ± 0.74 ^a^	26.98 ± 1.59 ^a^	26.22 ± 2.23 ^a^	3.99 ± 0.55 ^a^	3.47 ± 0.33 ^a^
	10	41.33 ± 3.45 ^a^	41.67 ± 2.72 ^a^	−17.25 ± 0.83 ^b^	−16.69 ± 1.51 ^a^	26.64 ± 1.56 ^a^	26.79 ± 2.26 ^a^	3.29 ± 0.52 ^a^	3.97 ± 2.15 ^a^
	14	41.03 ± 2.14 ^a^	41.99 ± 2.74 ^a^	−17.23 ± 1.20 ^b^	−17.71 ± 1.01 ^b^	26.11 ± 1.74 ^a^	27.36 ± 1.52 ^a^	2.93 ± 0.42 ^a^	4.45 ± 3.05 ^a^
Red cabbage	0	27.70 ± 2.06 ^a^	27.31 ± 1.20 ^b^	33.94 ± 3.35 ^a^	33.22 ± 3.07 ^a^	−8.7 ± 1.38 ^a^	−8.95 ± 0.99 ^d^	0	1.81 ± 0.41 ^c^
	3	27.81 ± 1.66 ^a^	34.63 ± 2.48 ^a^	32.38 ± 2.57 ^ab^	23.15 ± 3.65 ^b^	−8.72 ± 1.67 ^a^	−5.52 ± 3.05 ^c^	2.43 ± 1.34 ^b^	13.71 ± 2.52 ^b^
	5	27.59 ± 2.16 ^a^	35.10 ± 2.67 ^a^	29.52 ± 2.81 ^bc^	20.36 ± 2.31 ^bc^	−8.45 ± 1.67 ^a^	0.02 ± 2.81 ^b^	4.83 ± 1.95 ^a^	18.25 ± 0.3 ^ab^
	7	27.92 ± 1.88 ^a^	35.40 ± 3.28 ^a^	28.35 ± 2.90 ^bc^	18.73 ± 2.64 ^c^	−9.48 ± 1.93 ^a^	0.39 ± 2.43 ^ab^	6.62 ± 3.07 ^a^	19.07 ± 3.09 ^a^
	10	28.18 ± 1.55 ^a^	34.98 ± 2.57 ^a^	27.56 ± 4.31 ^c^	20.19 ± 1.70 ^bc^	−8.38 ± 2.30 ^a^	1.81 ± 1.21 ^ab^	6.98 ± 3.44 ^a^	18.77 ± 1.00 ^ab^
	14	26.62 ± 2.22 ^a^	34.34 ± 3.22 ^a^	26.36 ± 3.27 ^c^	18.19 ± 3.25 ^c^	−8.12 ± 1.34 ^a^	2.95 ± 1.90 ^a^	8.13 ± 2.17 ^a^	20.78 ± 1.15 ^a^
Carrot	0	53.77 ± 2.47 ^c^	53.61 ± 2.53 ^c^	26.67 ± 2.89 ^c^	26.40 ± 2.57 ^c^	43.11 ± 3.86 ^a^	43.68 ± 3.48 ^a^	0	3.19 ± 0.57 ^b^
	3	56.45 ± 1.89 ^b^	56.19 ± 1.93 ^b^	28.61 ± 1.63 ^b^	28.32 ± 2.16 ^bc^	42.64 ± 1.82 ^a^	42.93 ± 2.25 ^a^	4.51 ± 0.85 ^b^	3.21 ± 0.05 ^b^
	5	56.43 ± 2.17 ^b^	56.06 ± 1.47 ^b^	28.63 ± 1.86 ^b^	28.49 ± 0.95 ^b^	42.31 ± 2.24 ^a^	42.08 ± 1.95 ^a^	4.79 ± 0.16 ^ab^	4.11 ± 0.37 ^b^
	7	56.78 ± 1.78 ^b^	56.71 ± 1.39 ^b^	28.66 ± 0.99 ^b^	28.39 ± 1.15 ^bc^	42.59 ± 1.38 ^a^	42.67 ± 1.49 ^a^	4.05 ± 0.49 ^b^	3.69 ± 0.68 ^b^
	10	57.44 ± 1.04 ^b^	57.16 ± 1.74 ^b^	28.97 ± 1.55 ^b^	28.51 ± 0.80 ^b^	42.02 ± 2.05 ^a^	42.10 ± 1.53 ^a^	5.12 ± 1.38 ^ab^	4.88 ± 0.72 ^ab^
	14	60.08 ± 1.15 ^a^	59.98 ± 1.28 ^a^	33.75 ± 1.98 ^a^	32.57 ± 1.48 ^a^	43.89 ± 1.81 ^a^	42.86 ± 1.89 ^a^	8.07 ± 0.41 ^a^	7.53 ± 1.49 ^a^
Chicken breast cube	0	80.74 ± 1.15 ^a^	80.68 ± 1.20 ^a^	2.14 ± 0.51 ^a^	2.05 ± 0.40 ^a^	15.85 ± 0.74 ^a^	15.89 ± 0.78 ^b^	0	0.37 ± 0.08 ^c^
	3	80.68 ± 0.74 ^a^	80.63 ± 1.08 ^a^	1.45 ± 0.49 ^b^	1.29 ± 0.41 ^b^	15.90 ± 0.65 ^a^	15.88 ± 0.82 ^b^	0.79 ± 0.14 ^a^	1.17 ± 0.01 ^ab^
	5	80.64 ± 1.30 ^a^	80.30 ± 1.14 ^a^	1.35 ± 0.43 ^b^	1.30 ± 0.44 ^b^	15.98 ± 0.57 ^a^	15.94 ± 0.88 ^b^	0.85 ± 0.01 ^a^	1.01 ± 0.1 ^abc^
	7	80.33 ± 1.19 ^a^	80.87 ± 1.33 ^a^	1.36 ± 0.61 ^b^	1.33 ± 0.42 ^b^	15.95 ± 0.68 ^a^	15.93 ± 0.72 ^b^	1.2 ± 0.46 ^a^	0.93 ± 0.38 ^bc^
	10	80.39 ± 1.39 ^a^	80.28 ± 0.93 ^a^	1.10 ± 0.42 ^b^	1.04 ± 0.49 ^b^	16.01 ± 1.05 ^a^	16.31 ± 1.04 ^ab^	1.33 ± 0.43 ^a^	1.51 ± 0.21 ^ab^
	14	80.77 ± 1.22 ^a^	81.10 ± 1.15 ^a^	1.12 ± 0.61 ^b^	1.15 ± 0.32 ^b^	16.22 ± 0.75 ^a^	16.61 ± 0.84 ^a^	1.16 ± 0.45 ^a^	1.61 ± 0.24 ^a^

ADCPT, ADCP treated. The values are mean and standard deviation (*n* = 4). Means followed by the same lowercase letter in a column are not significantly different for each content in chicken breast salad (*p* > 0.05).

**Table 3:** Effects of atmospheric dielectric barrier discharge cold plasma (ADCP) treatment at 24 kV for 2 min on the antioxidant capacity of lettuce, red cabbage, and carrot during storage at 4 °C.

Storage Time (d)	Lettuce				Red Cabbage				Carrot			
	DPPH		ABTS		DPPH		ABTS		DPPH		ABTS	
	Untreated	ADCPT	Untreated	ADCPT	Untreated	ADCPT	Untreated	ADCPT	Untreated	ADCPT	Untreated	ADCPT
0	21.6 ± 2.6 ^a^	20.9 ± 1.9 ^a^	65.8 ± 5.9 ^a^	66.4 ± 5.9 ^a^	19.6 ± 1.7 ^a^	19.2 ± 2.0 ^a^	76.8 ± 1.7 ^a^	77.0 ± 2.3 ^a^	37.3 ± 1.9 ^a^	37.1 ± 0.9 ^a^	47.9 ± 1.0 ^a^	47.7 ± 1.0 ^a^
3	14.3 ± 1.5 ^b^	14.8 ± 1.2 ^b^	65.5 ± 3.2 ^a^	65.7 ± 3.1 ^a^	18.4 ± 1.0 ^a^	18.2 ± 1.2 ^a^	77.0 ± 1.9 ^a^	76.9 ± 1.7 ^a^	36.8 ± 0.7 ^a^	36.2 ± 1.1 ^a^	47.7 ± 0.5 ^a^	47.9 ± 0.9 ^a^
5	14.2 ± 1.2 ^b^	14.3 ± 1.0 ^b^	66.5 ± 3.0 ^a^	65.2 ± 3.4 ^a^	18.5 ± 1.0 ^a^	18.3 ± 1.2 ^a^	77.2 ± 0.7 ^a^	76.9 ± 1.3 ^a^	36.2 ± 0.9 ^a^	36.9 ± 0.5 ^a^	47.8 ± 1.6 ^a^	47.7 ± 1.7 ^a^
7	14.3 ± 1.7 ^b^	14.8 ± 1.1 ^b^	66.4 ± 3.6 ^a^	66.0 ± 3.0 ^a^	17.5 ± 1.3 ^b^	17.5 ± 0.8 ^b^	77.3 ± 0.9 ^a^	77.0 ± 0.8 ^a^	36.7 ± 0.5 ^a^	36.5 ± 1.1 ^a^	46.2 ± 0.9 ^a^	46.7 ± 0.6 ^a^
10	14.4 ± 1.5 ^b^	14.7 ± 1.9 ^b^	65.8 ± 2.5 ^a^	66.4 ± 3.0 ^a^	17.2 ± 1.3 ^b^	17.7 ± 0.7 ^b^	77.3 ± 1.0 ^a^	77.2 ± 1.1 ^a^	35.6 ± 0.5 ^b^	35.6 ± 1.1 ^b^	45.9 ± 0.7 ^b^	45.9 ± 0.9 ^b^
14	14.6 ± 1.2 ^b^	14.6 ± 1.1 ^b^	66.1 ± 3.1 ^a^	66.4 ± 1.6 ^a^	17.2 ± 1.6 ^b^	17.1 ± 1.4 ^b^	77.3 ± 0.8 ^a^	77.0 ± 1.2 ^a^	34.6 ± 0.5 ^b^	34.9 ± 0.5 ^b^	45.7 ± 0.6 ^b^	45.3 ± 0.7 ^b^

ADCPT, ADCP treated. The values are mean and standard deviation (*n* = 8). Means followed by the same lowercase letter in a column are not significantly different at 4 °C (*p* > 0.05).

**Table 4:** Effects of atmospheric dielectric barrier discharge cold plasma (ADCP) treatment at 24 kV for 2 min on sensory attributes of chicken breast salad.

Samples		Sensory Attributes		
		Color	Smell	Appearance
Unstored	Untreated	6.5 ± 1.8 a	5.3 ± 1.6 a	6.0 ± 1.7 a
	ADCPT	6.2 ± 1.6 a	5.1 ± 1.4 a	5.9 ± 1.7 a
3-day storage at 4 °C	Untreated	6.1 ± 1.8 a	5.5 ± 1.3 a	6.2 ± 1.7 a
	ADCPT	4.9 ± 1.6 b	5.0 ± 1.3 a	5.1 ± 1.8 b

ADCPT, ADCP-treated. The values are means and standard deviations (*n* = 40). Means followed by the same lowercase letter in a column are not significantly different on each storage day (*p* > 0.05).

## Data Availability

The datasets generated for this study are available on request to the corresponding author.
